# American Society of Orthodontists

**Published:** 1902-11

**Authors:** 


### AMERICAN SOCIETY OF ORTHODONTISTS.

THIS new Society, devoted to the consideration of orthodontia and related conditions, opened its second annual session at the Colonnade Hotel, Philadelphia, October 8, and extended through three days.

The attendance was not large, ranging from thirty to forty, but what it lacked in numbers it made up in interest in the work. The division of this branch of dentistry into a specialty has been too recent to expect an extended interest, and numbers could not be anticipated and, perhaps, not desired. Then, again, there is a large body of dentists, skilled orthodontists, who do not favor an organization specially devoted to this subject.

The present meeting, so far as the writer is capable of judging from a limited observation, entertained a broad comprehension of the subject, and the papers, upon the whole, were interesting, although condensation would have materially improved one or two.

Orthodontia, while old as dentistry itself, is still in the early stages of development so far as the determination of the principles governing it are concerned. No better illustration of these has ever been attempted than in the monumental work of Farrar, but even with this as a guide there is much still to be desired.

It was gratifying to the writer to observe that the active members were nearly all comparatively young in years. Their enthusiasm upon the subject speaks well for progress in this important branch of dental labor, providing there is not a too close adherence to special methods or narrow conceptions.

Dr. Angle presided over the meetings, which were conducted with dignity due to the subject.

Among those present and taking an active interest was Dr. Grevers, of Holland, on a short visit to this country. Dr. Grevers is much interested in dental education and the work which has occupied the attention of the Fédèration Dentaire Internationale which recently met in Stockholm.

